# Mode of birth and medical interventions among women at low risk of complications: A cross-national comparison of birth settings in England and the Netherlands

**DOI:** 10.1371/journal.pone.0180846

**Published:** 2017-07-27

**Authors:** Ank de Jonge, Lilian Peters, Caroline C. Geerts, Jos J. M. van Roosmalen, Jos W. R. Twisk, Peter Brocklehurst, Jennifer Hollowell

**Affiliations:** 1 Department of Midwifery Science, AVAG and Amsterdam Public Health research institute, VU University Medical Center at Amsterdam, Amsterdam, the Netherlands; 2 Athena Institute, VU University, Amsterdam, the Netherlands; 3 Department of Clinical Epidemiology and Biostatistics, VU University Medical Center Amsterdam, Amsterdam, the Netherlands; 4 National Perinatal Epidemiology Unit (NPEU), University of Oxford, Oxford, United Kingdom; 5 Birmingham Clinical Trials Unit, University of Birmingham, Birmingham, United Kingdom; Stellenbosch University, SOUTH AFRICA

## Abstract

**Objectives:**

To compare mode of birth and medical interventions between broadly equivalent birth settings in England and the Netherlands.

**Methods:**

Data were combined from the Birthplace study in England (from April 2008 to April 2010) and the National Perinatal Register in the Netherlands (2009). Low risk women in England planning birth at home (16,470) or in freestanding midwifery units (11,133) were compared with Dutch women with planned home births (40,468). Low risk English women with births planned in alongside midwifery units (16,418) or obstetric units (19,096) were compared with Dutch women with planned midwife-led hospital births (37,887).

**Results:**

CS rates varied across planned births settings from 6.5% to 15.5% among nulliparous and 0.6% to 5.1% among multiparous women. CS rates were higher among low risk nulliparous and multiparous English women planning obstetric unit births compared to Dutch women planning midwife-led hospital births (adjusted (adj) OR 1.89 (95% CI 1.64 to 2.18) and 3.66 (2.90 to 4.63) respectively).

Instrumental vaginal birth rates varied from 10.7% to 22.5% for nulliparous and from 0.9% to 5.7% for multiparous women. Rates were lower in the English comparison groups apart from planned births in obstetric units. Transfer, augmentation and episiotomy rates were much lower in England compared to the Netherlands for all midwife-led groups. In most comparisons, epidural rates were higher among English groups.

**Conclusions:**

When considering maternal outcomes, findings confirm advantages of giving birth in midwife-led settings for low risk women. Further research is needed into strategies to decrease rates of medical intervention in obstetric units in England and to reduce rates of avoidable transfer, episiotomy and augmentation of labour in the Netherlands.

## Introduction

Although most women in high income countries give birth in obstetric units, in some countries women at low risk of complications (‘low risk women’) can choose to give birth at home or in midwifery units.[1,2] In 2012, 2% of women in England gave birth at home and 11% in midwifery units[3]; in the Netherlands 16% gave birth at home and 13% in hospital assisted by primary care midwives.[4,5] Large studies in England and the Netherlands have shown low rates of adverse outcomes among all low risk women, although higher rates of adverse perinatal oucomes were found among nulliparous women in England planning birth at home versus in obstetric units (9.3 versus 5.3 per 1000 births, adjusted odds ratio 1.75, 95% CI 1.07 to 2.86).[6] Rates of obstetric interventions, however, were higher among births planned in obstetric units compared to those planned at home or in midwifery units.[6] In the Netherlands, studies have shown lower intervention rates among women in midwife-led care who planned birth at home versus in hospital. [7–9]

Although obstetric interventions can be life-saving, they have potential side effects. For example, women who had caesarean section (CS) are more likely to suffer from severe acute maternal morbidity, such as major blood loss and thrombo-embolism, infection or adhesions.[10–13] During pregnancies following CS, rates of unexplained stillbirth are increased[14] and uterine scars may rupture.[15–17] Other medical interventions, such as instrumental vaginal births and oxytocin use or epidural anaesthesia, are also associated with potential adverse side effects, such as postpartum haemorrhage.[18,19]

There are large variations in rates of obstetric interventions between and within countries.[20] For example, CS rates in Europe vary from 14.8% in Iceland to 52.2% in Cyprus.[20] It is important to examine factors that explain these variations to inform strategies to optimise rates across the world. Some of these factors are related to characteristics of the maternity care system.[21]

There is evidence that midwife-led birth settings are associated with lower intervention rates among low-risk women but between country differences in intervention rates in different birth settings have not been explored.[6,7,22–26] Such cross-national comparisons have the potential to shed light on factors that influence intervention rates and may suggest ways in which maternity care systems can be improved. England and the Netherlands are particularly suitable for such comparisons since both countries have well established midwife-led care, although the models of care differ between the two countries. Additionally, proportions of women opting for midwife-led birth settings differ in the two countries which may also influence intervention rates. An exploratory analysis in England found that intervention rates were higher in planned obstetric unit births compared to births in midwife-led settings in areas where higher proportions of women planned birth in midwife-led settings.[27]

The aim of this study was to use individual client data to compare intra-partum caesarean section and instrumental vaginal birth rates among low risk women between broadly equivalent birth settings in England and the Netherlands. Secondly, we wanted to explore whether planned home birth in the two countries is associated with a similar change in CS rate compared to planned midwife-led hospital birth. Thirdly, we aimed to compare other medical intervention rates between birth settings in England and the Netherlands.

## Methods

### Study population

For this cohort study, data were combined from the English Birthplace Study (BPS) and the national Dutch Perinatal Register (PRN). As described more fully elsewhere, BPS data were collected from 84%-97% of freestanding and alongside midwifery units and trusts providing home birth services in England and from a stratified random sample of 36 obstetric units between April 2008 and April 2010.[6] Duration of data collection varied between units and trusts.[6]

In the Netherlands, perinatal registration data are collected in three separate databases: one for primary midwife-led care (perinatal database-1), one for secondary obstetric care (perinatal database-2) and one for neonatal care (neonatal database).[28] About 99% of primary care practices and 100% of obstetric care practices provide data for the PRN. All academic hospitals and about 50% of peripheral hospitals provide data for the neonatal database. These databases have been combined into one national perinatal database via a validated linkage method.[29]

For the original Birthplace study, research ethics committee approval was obtained from the Berkshire Research Ethics Committee (MREC ref 07/H0505/151) and the ethical committee of VU University Medical Center confirmed that ethical approval was not necessary for this study in the Netherlands (ref no 11/399).

Our study population comprised women with singleton, term pregnancies (37–41+6 weeks gestation)[30] with spontaneous onset of labour, planning spontaneous vaginal birth and without obstetric or medical risk factors during pregnancy. We only included women in the BPS (subsequently referred to as ‘English women’) if, prior to the onset of labour, they did not have any medical or obstetric risk factors listed in the NICE guideline on intrapartum care.[31] All Dutch women starting labour in midwife-led care in 2009 and without an indication for giving birth in hospital were included (subsequently referred to as ‘Dutch women’). Indications for referral to obstetrician-led care are laid down in the Dutch obstetric indication list.[32]

In both countries, we excluded women who had no antenatal care. In England, planned place of birth was defined as intended place of birth at the start of face-to-face care in labour whereas in the Netherlands planned place of birth would have been recorded by midwives during pregnancy. Unplanned home births in England, which are generally unattended, were excluded. In the Netherlands, midwives always visit women in labour at home, regardless of where they plan to give birth; it is not unusual for women with planned hospital birth to give birth at home because their labour is progressing too fast to move to hospital or because they change their mind during labour.[33] Therefore, unplanned home births are generally attended by a midwife in the Netherlands and do not necessarily increase risk and these were not excluded in the Netherlands for the primary analyses.

Lists of indications for obstetric care are similar in both countries although some indications differ. For example, in the Netherlands uncomplicated anaemia is not a reason for obstetric care, whereas in England it is. In the Netherlands, body mass index (BMI) is not mentioned in the indication list. However, a guideline developed by the Dutch Society of Obstetrics and Gynaecology recommends obstetric care when pre-pregnancy BMI is over 40 whereas the cut-off point in the NICE guideline is 35.[31,34] Meconium stained liquor is always an indication for obstetric care in the Netherlands whereas uncomplicated, light meconium stained liquor is not a reason for transfer of care in England.

### Comparison groups

Groups of low risk women were compared based on planned place of birth at the start of care in labour regardless of where birth took place. In England, low risk women can choose to give birth in midwife-led care at home, in freestanding midwifery units (‘freestanding unit’) or in alongside midwifery units (‘alongside unit’) or in obstetrician-led care in obstetric units. Although midwives provide most care in obstetric units, obstetricians are responsible. In the Netherlands, low risk women plan their births at home or in hospital. At the time of the study, midwifery units hardly existed and most births in these units would have been recorded as hospital births. In both countries, women are referred to obstetrician-led care in hospital if complications occur during labour and midwives who have provided care up to the point of transfer do not continue to provide care.[28,35]

Giving birth in freestanding units in England is somewhat comparable to home birth because transport is required if obstetric care is needed. Women giving birth in alongside or obstetric units are comparable to the extent that midwives provide most care unless complications develop, and if transfer of care is required from an alongside unit, this will only involve transport within the same building or at the same site. We compared women planning births: (a) at home in England versus at home in the Netherlands, (b) in freestanding units in England versus at home in the Netherlands, (c) in alongside units in England versus in hospital in the Netherlands (midwife-led), and (d) in obstetric units (obstetrician-led) in England versus in hospital in the Netherlands (midwife-led).

### Study outcomes and confounders

Primary outcomes were unplanned, intrapartum caesarean section, instrumental vaginal birth (ventouse or forceps) and operative birth (composite of caesarean section and instrumental vaginal birth). Secondary outcomes were oxytocin augmentation, regional analgesia (epidural or spinal), transfer to obstetrician-led care during labour or immediately after birth, third or fourth degree perineal trauma, episiotomy and oxytocin during the third stage of labour; the latter included any oxytocin given after birth of the baby in the Netherlands and was defined as active management of labour in the BPS.

Potential confounders were defined in a similar way as much as possible in both datasets. Ethnicity (based on country of birth of pregnant women and their parents) is not recorded uniformly by midwives and therefore we dichotomized ethnic background as Dutch/ White British or non-Dutch/ non-White British. Socio-economic position in both countries was based on area indices of deprivation based on women’s postal codes and defined as low (below the 25^th^ percentile (P25), medium (P25-P75) or high (> P75).

BMI is not recorded in the Dutch national perinatal register. To explore the influence of BMI on the association between planned place of birth and caesarean section, we used data from the Dutch Deliver study. This was a multi-center prospective study into quality and provision of primary midwifery care in the Netherlands, conducted between September 2009 and December 2010.[36] BMI was used as recorded in a questionnaire filled in by women or, if missing, in their maternity care notes.

### Data-analysis

We compared outcomes among women in the groups as outlined earlier based on planned place of birth. We also compared differences in CS rates between women in England planning birth at home versus in alongside units with differences in CS rates between women in the Netherlands planning birth at home versus in hospital (midwife-led) because we assumed that these settings were most comparable.

Results were stratified for nulliparous and multiparous women. Unadjusted and adjusted odds ratios (ORs) were calculated using logistic regression. For primary outcomes 95% confidence intervals (CIs) were calculated and for secondary outcomes 99% CI’s. Results were adjusted for maternal age in three categories (< 25, 25–35, > 35 years), gestational age in completed weeks (37–37+6, 38–40+6, 41–41+6) and socio-economic position and ethnic background as defined earlier.

Robust variance estimation was used to account for data clustering within trusts and units in England, and within midwifery practices in the Netherlands. Probability weights were used to account for differences in the probability of women being selected for inclusion arising from differences in each unit or trust’s period of participation and stratum-specific probabilities of selection of obstetric units. For PRN data all observations had the same weight (1) because all eligible births in 2009 were included. Multivariable analyses were performed using complete cases because less than 5% of records had missing data. Analyses were performed using Stata 12 and SPSS Statistics 22.0 (SPSS inc. Chicago, Illinois).

#### Additional analyses

The Dutch perinatal register does not have a variable indicating start of labour in primary or secondary care but this variable is created based on information from perinatal databases-1 and -2 and this information is not always consistent. We conducted sensitivity analyses for the differences in main outcomes after excluding women with discrepancies in information that is used to define start of labour and for CS after excluding women in the Netherlands who planned birth in hospital but gave birth at home. To explore whether differences in BMI may have influenced results[37], we compared CS rates between women in the BPS and women from the Deliver study(36) and added BMI as a confounder (BMI not recorded, < 18.4, 18.5–24.9, 25–29.9, 30–35).

## Results

### Study population

**Fig 1:**
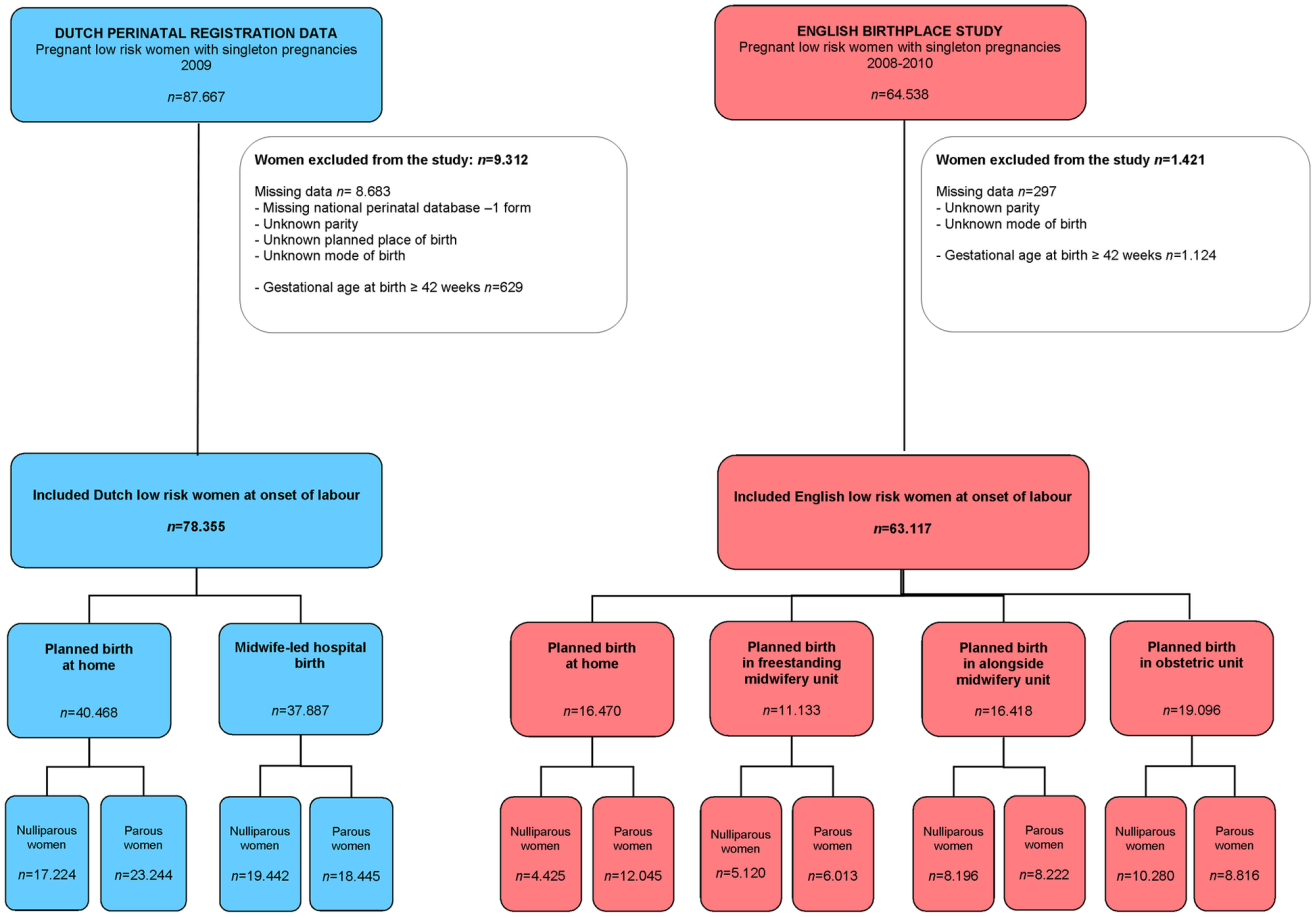
Flow diagram of participants in the study.

From the BPS in England we included 16,470 low risk women planning birth at home, 11,133 in freestanding midwifery units, 16,418 in alongside midwifery units and 19,096 in obstetric units (Fig 1). From the Dutch PRN data we included 40,468 women planning birth at home and 37,887 in hospital (midwife-led).

There were differences in the characteristics of women giving birth in different settings in the two countries (Table 1). For example, women planning home birth in England were more likely to be multiparous than women planning home birth in the Netherlands.

**Table 1:** Baseline characteristics of low risk women at the start of labour by country specific birth setting.

Baseline characteristics	Planned home birth NL	Planned home birth England	Planned birth in freestanding midwifery Unit England	Midwife-led hospital birth NL	Planned birth in alongside midwifery unit England	Planned birth in obstetric unit England
	*n* = 40,468	*n* = 16,470	*n* = 11,133	*n* = 37,887	*n* = 16,418	*n* = 19,096
**Parity, n (%)**						
Nulliparous	17,224 (42.6)	4,425 (26.9)	5,120 (46.0)	19,442 (51.3)	8,196 (49.9)	10,280 (53.8)
Multiparous	23,244 (57.4)	12,045 (73.1)	6,013 (54.0)	18,445 (48.7)	8,222 (50.1)	8,816 (46.2)
**Gestational age (completed wks), n (%)**						
37–37+6 weeks	1,588 (3.9)	376 (2.3)	314 (2.8)	1,702 (4.5)	473 (2.9)	715 (3.7)
38–40+6 weeks	30,246 (74.7)	12,236 (74.3)	8,001 (71.9)	28,642 (75.6)	12,151 (74.0)	13,480 (70.6)
41–41+6 weeks	8,634 (21.3)	3,858 (23.4)	2,818 (25.3)	7,543 (19.9)	3,794 (23.1)	4,901 (25.7)
**Maternal age, n (%)**						
<25 years	4,248 (10.5)	1,895 (11.5)	2,773 (24.9)	5,446 (14.4)	4,463 (27.2)	5,561 (29.1)
25–35 years	29,006 (71.7)	9,976 (60.6)	6,429 (57.7)	25,426 (67.1)	9,429 (57.4)	10,466 (54.8)
>35 years	7,213 (17.8)	4,569 (27.7)	1,917 (17.2)	7,015 (18.5)	2,488 (15.2)	3,047 (16)
Missing	1 (0)	30 (0.2)	14 (0.1)	-	38 (0.2)	22 (0.1)
**Ethnic background, n (%)**						
Dutch/White British	36,414 (90.0)	15,585 (94.6)	10,191 (91.5)	25,934 (68.5)	13,227 (80.6)	15,568 (81.6)
Non-Dutch/non-White British	3,909 (9.7)	865 (5.3)	937 (8.4)	11,718 (30.9)	3,156 (19.2)	3,503 (18.3)
Missing	145 (0.4)	20 (0.1)	5 (0.04)	235 (0.6)	35 (0.2)	25 (0.1)
**Socio economic position, n (%)**						
Low <P25	10,805 (26.7)	3,283 (19.9)	2,183 (19.6)	13,438 (35.5)	5,273 (32.1)	5,947 (31.1)
Medium P25-P75	19,623 (48.5)	8,631 (52.4)	5,851 (52.6)	14,880 (39.3)	8,007 (48.8)	9,101 (47.7)
High >P75	9,178 (22.7)	4,444 (27.0)	3,068 (27.6)	8,602 (22.7)	3,090 (18.8)	3,926 (20.6)
Missing	862 (2.1)	112 (0.7)	31 (0.3)	967 (2.6)	48 (0.3)	122 (0.6)
**Birthweight, n (%)**						
<2500 grams	241 (0.6)	85 (0.5)	100 (0.9)	321 (0.9)	159 (1)	273 (1.4)
2500–3499 grams	17,333 (42.8)	7,467 (45.3)	5,707 (51.3)	18,211 (48.1)	8,792 (53.6)	10,343 (54.2)
3500–3999 grams	15,575 (38.5)	6,266 (38.0)	3,961 (35.6)	13,624 (36.0)	5,571 (33.9)	6,249 (32.7)
>4000-	7,307 (18.1)	2,604 (15.8)	1,360 (12.2)	5,710 (15.1)	1,853 (11.3)	2,202 (11.5)
Missing	12 (0.03)	48 (0.3)	5 (0.04)	11 (0.03)	43 (0.3)	29 (0.2)

### Operative birth

CS rate in our study population varied across birth settings from 6.5% to 15.5% among nulliparous and 0.6% to 5.1% among multiparous women (Table 2). There were no significant differences in CS rates between English women planning birth at home or in freestanding units compared to Dutch women planning home birth. Nulliparous and multiparous English women planning obstetric unit birth had higher rates of CS compared to Dutch women planning midwife-led hospital birth (adjusted (adj) OR and 95% CI 1.89 (1.64–2.18) and 3.66 (2.90–4.63) respectively). English multiparous women planning birth in alongside units in England had lower CS rates compared to Dutch multiparous women planning midwife-led hospital births (adj OR 0.70, 95% CI 0.48 to 1.00).

**Table 2:** Planned place of birth and rate of caesarean section.

Planned place of birth	No of events	Incidence of caesarean section/ 100^ (95% CI)	Odds ratio (95% CI)	
			Unadjusted	Adjusted*
**Nulliparous women**				
Home NL	1,279	7.4 (7.0–7.9)	1.00	1.00
Home England	355	8.4 (7.1–9.7)	1.14 (0.95–1.36)	1.05 (0.88–1.26)
Freestanding midwifery unit England	345	6.5 (5.5–7.5)	0.87 (0.73–1.03)	0.93 (0.79–1.09)
Midwife-led hospital birth NL	1,806	9.3 (8.7–9.8)	1.00	1.00
Alongside midwifery unit England	619	7.6 (6.5–8.8)	**0.81 (0.68–0.96)**	0.86 (0.70–1.04)
Obstetric unit England	1,575	15.5 (13.9–17.1)	**1.79 (1.56–2.06)**	**1.89 (1.64–2.18)**
**Multiparous women**				
Home NL	188	0.8 (0.7–0.9)	1.00	1.00
Home England	80	0.6 (0.5–0.8)	0.77 (0.57–1.04)	0.75 (0.55–1.02)
Freestanding midwifery unit England	44	0.7 (0.5–0.9)	0.89 (0.62–1.27)	0.91 (0.63–1.32)
Midwife-led hospital birth NL	271	1.5 (1.3–1.7)	1.00	1.00
Alongside midwifery unit England	87	1.0 (0.7–1.3)	**0.70 (0.49–0.98)**	**0.70 (0.48–1.00)**
Obstetric unit England	446	5.1 (4.1–6.1)	**3.61 (2.83–4.61)**	**3.66 (2.90–4.63)**

^Weighted to reflect each unit’s separate duration of participation and probability of being sampled; confidence intervals take account of the clustered nature of the data.

* Adjusted for maternal age, gestational age, socioeconomic position and ethnic background.

Instrumental vaginal birth rates varied from 10.7% to 22.5% for nulliparous and from 0.9% to 5.7% for multiparous women (Table 3). Nulliparous English women planning birth at home or in freestanding units had lower rates of instrumental vaginal birth compared to Dutch women planning home birth (adj OR 0.63, 95% CI 0.56 to 0.70 and 0.60, 0.50 to 0.72 respectively). Nulliparous English women planning birth in alongside units in England had lower rates compared to Dutch women planning hospital birth (adj OR0.84, 95% CI 0.71 to 0.98). English women planning obstetric unit birth had higher rates compared to Dutch women planning midwife-led hospital birth (adj OR 1.29, 95% CI 1.09 to 1.52 for nulliparous and 2.75, 95% CI 2.26 to 3.35 for multiparous women).

**Table 3:** Planned place of birth and rate of instrumental vaginal birth (ventouse or forceps).

Planned place of birth	No of events	Incidence of instrumental vaginal birth/ 100 (95% CI)^	Odds ratio (95% CI)	
			Unadjusted	Adjusted*
**Nulliparous women**				
Home NL	2,946	17.1 (16.4–17.8)	1.00	1.00
Home England	571	12.4 (11.3–13.5)	**0.69 (0.61–0.77)**	**0.63 (0.56–0.70)**
Freestanding midwifery unit England	602	10.7 (9.0–12.5)	**0.58 (0.48–0.70)**	**0.60 (0.50–0.72)**
Midwife-led hospital birth NL	3,622	18.6 (17.9–19.4)	1.00	1.00
Alongside midwifery unit England	1,289	16.0 (14.0–17.9)	**0.83 (0.71–0.97)**	**0.84 (0.71–0.98)**
Obstetric unit England	2,251	22.5 (19.9–25.1)	**1.27 (1.09–1.48)**	**1.29 (1.09–1.52)**
**Multiparous women**				
Home NL	232	1.0 (0.9–1.1)	1.00	1.00
Home England	109	0.9 (0.7–1.1)	0.90 (0.70–1.15)	0.90 (0.70–1.16)
Freestanding midwifery unit England	69	1.0 (0.7–1.3)	1.02 (0.72–1.45)	1.01 (0.70–1.44)
Midwife-led hospital birth NL	393	2.1 (1.9–2.4)	1.00	1.00
Alongside midwifery unit England	188	2.4 (1.9–3.0)	1.15 (0.90–1.46)	1.14 (0.89–1.46)
Obstetric unit England	491	5.7 (4.8–6.6)	**2.76 (2.27–3.36)**	**2.75 (2.26–3.35)**

^Weighted to reflect each unit’s separate duration of participation and probability of being sampled; confidence intervals take account of the clustered nature of the data.

* Adjusted for maternal age, gestational age, socioeconomic position and ethnic background.

Rates of operative births (CS and instrumental vaginal births combined) were 17.2% to 38.0% for nulliparous and 1.5% to 10.8% for multiparous women (Table 4). Patterns were similar to those for instrumental vaginal births.

**Table 4:** Planned place of birth and rate of operative births (caesarean section or instrumental vaginal birth).

Planned place of birth	No of events	Incidence of operative births/ 100 (95% CI)^	Odds ratio (95% CI)	
			Unadjusted	Adjusted*
**Nulliparous women**				
Home NL	4,225	24.5 (23.7–25.4)	1.00	1.00
Home England	926	20.8 (19.1–22.5)	**0.81 (0.72–0.90)**	**0.73 (0.65–0.81)**
Freestanding midwifery unit England	947	17.2 (15.0–19.5)	**0.64 (0.55–0.75)**	**0.67 (0.57–0.78)**
Midwife-led hospital birth NL	5,428	27.9 (26.9–28.9)	1.00	1.00
Alongside midwifery unit England	1,908	23.6 (21.2–26.0)	**0.80 (0.69–0.92)**	**0.82 (0.70–0.97)**
Obstetric unit England	3,826	38.0 (35.4–40.7)	**1.58 (1.40–1.79)**	**1.66 (1.47–1.88)**
**Multiparous women**				
Home NL	420	1.8 (1.6–2.0)	1.00	1.00
Home England	189	1.5 (1.3–1.8)	0.84 (0.69–1.02)	0.83 (0.68–1.02)
Freestanding midwifery unit England	113	1.7 (1.3–2.2)	0.96 (0.72–1.29)	0.96 (0.71–1.31)
Midwife-led hospital birth NL	664	3.3 (3.3–3.9)	1.00	1.00
Alongside midwifery unit England	275	3.5 (2.7–4.2)	0.96 (0.76–1.22)	0.96 (0.75–1.22)
Obstetric unit England	937	10.8 (9.4–12.2)	**3.23 (2.73–3.84)**	**3.26 (2.76–3.84)**

^Weighted to reflect each unit’s separate duration of participation and probability of being sampled; confidence intervals take account of the clustered nature of the data.

* Adjusted for maternal age, gestational age, socioeconomic position and ethnic background.

Table 5 shows comparisons of CS rates between groups within the Netherlands and within England. For nulliparous women there was no significant difference in CS rate between English women planning home birth compared to women planning birth in alongside units whereas Dutch nulliparous women planning home birth had lower CS rates compared to those with planned midwife-led hospital births (adj OR 0.83, 95% CI 0.77 to 0.90). For multiparous women, differences in CS rate between English women planning home birth compared to women planning birth in alongside units were similar to differences for Dutch women planning home birth compared to those planning midwife-led hospital birth (adj OR 0.54, 95% CI 0.36 to 0.81 and adj OR 0.61, 95% CI 0.50 to 0.76 respectively).

**Table 5:** Comparison of difference in CS rate between planned home birth and planned hospital birth in the Netherlands with the difference in CS rate between planned home and planned birth in an alongside midwifery unit in England.

Planned place of birth	No of events	Incidence of caesarean section/ 100^ (95% CI)	Odds ratio (95% CI)	
			Unadjusted	Adjusted*
**Nulliparous women**				
Midwife-led hospital birth NL	1,806	9.3 (8.7–9.8)	1.00	1.00
Home NL	1,279	7.4 (7.0–7.9)	**0.78 (0.72–0.85)**	**0.83 (0.77–0.90)**
Alongside unit/England	619	7.6 (6.5–8.8)	1.00	1.00
Home England	355	8.4 (7.1–9.7)	1.11 (0.88–1.39)	0.95 (0.73–1.22)
**Multiparous women**				
Midwife-led hospital birth NL	271	1.5 (1.3–1.7)	1.00	1.00
Home NL	188	0.8 (0.7–0.9)	**0.55 (0.45–0.67)**	**0.61 (0.50–0.76)**
Alongside unit England	87	1.0 (0.7–1.3)	1.00	1.00
Home England	80	0.6 (0.5–0.8)	**0.60 (0.40–0.91)**	**0.54 (0.36–0.81)**

^Weighted to reflect each unit’s separate duration of participation and probability of being sampled; confidence intervals take account of the clustered nature of the data.

* Adjusted for maternal age, gestational age, socioeconomic position and ethnic background.

### Augmentation of labour, regional anaesthesia and transfer

Rates of augmentation of labour varied from 13.6% to 37.7% among nulliparous and from 1.0% to 11.3% among multiparous women (Table 6). Rates were lower among English compared to Dutch women in all groups, although not significantly so for women planning birth in obstetric units.

**Table 6:** Augmentation with oxytocin, epidural or spinal analgesia and transfer of care.

Planned place of birth	No of events	Incidence/ 100 (99% CI)^	Odds ratio (99% CI)	
			Unadjusted	Adjusted*
**Nulliparous women** *Augmentation with oxytocin*				
Home NL	5,376	31.2 (29.9–32.5)	1.00	1.00
Home England	759	16.8 (14.8–18.8)	**0.44 (0.38–0.52)**	**0.41 (0.35–0.48)**
Freestanding midwifery unit England	753	13.6 (11.3–15.9)	**0.35 (0.28–0.43)**	**0.35 (0.28–0.43)**
Midwife-led hospital birth NL	7,321	37.7 (35.9–39.4)	1.00	1.00
Alongside midwifery unit England	1,459	17.6 (15.4–19.8)	**0.35 (0.30–0.42)**	**0.36 (0.30–0.42)**
Obstetric unit England	3,457	34.3 (31.0–37.6)	0.86 (0.73–1.02)	0.87 (0.74–1.02)
*Epidural or spinal analgesia*				
Home NL	2,468	16.5 (15.4–17.6)	1.00	1.00
Home England	995	22.3 (19.9–24.8)	**1.45 (1.23–1.71)**	**1.36 (1.15–1.60)**
Freestanding midwifery unit England	996	18.7 (16.1–21.3)	1.16 (0.96–1.40)	1.17 (0.96–1.42)
Midwife-led hospital birth NL	4,435	22.8 (21.2–24.4)	1.00	1.00
Alongside midwifery unit England	1,928	24.0 (20.8–27.1)	1.07 (0.88–1.29)	1.09 (0.89–1.34)
Obstetric unit England	4,139	41.9 (37.6–46.2)	**2.44 (2.00–2.97)**	**2.50 (2.06–3.04)**
*Transfer of care during labour or directly postpartum*				
Home NL	9,180	53.3 (51.7–54.9)	1.00	1.00
Home England	1,967	43.9 (40.9–46.8)	**0.68 (0.60–0.79)**	**0.64 (0.56–0.74)**
Freestanding midwifery unit England	1,846	34.3 (30.5–38.1)	**0.46 (0.38–0.55)**	**0.45 (0.38–0.55)**
Midwife-led hospital birth NL	11,753	60.5 (58.3–62.6)	1.00	1.00
Alongside midwifery unit England	3,270	39.8 (35.8–43.8)	**0.43 (0.36–0.52)**	**0.43 (0.35–0.52)**
**Multiparous women**				
*Augmentation Oxytocin/ syntocinon*				
Home NL	1,301	5.6 (5.2–6.0)	1.00	1.00
Home England	128	1.0 (0.7–1.3)	**0.17 (0.13–0.23)**	**0.18 (0.13–0.24)**
Freestanding midwifery unit England	94	1.4 (0.8–2.0)	**0.24 (0.15–0.36)**	**0.23 (0.15–0.36)**
Midwife-led hospital birth NL	2,083	11.3 (10.5–12.1)	1.00	1.00
Alongside midwifery unit England	195	2.4 (1.7–3.1)	**0.19 (0.14–0.27)**	**0.19 (0.14–0.26)**
Obstetric unit England	855	9.8 (8.0–11.5)	0.85 (0.68–1.06)	0.82 (0.66–1.02)
*Epidural or spinal analgesia*				
Home NL	328	1.4 (1.2–1.6)	1.00	1.00
Home England	348	2.8 (2.3–3.3)	**2.01 (1.56–2.57)**	**2.02 (1.57–2.61)**
Freestanding midwifery unit England	217	3.5 (2.7–4.3)	**2.51 (1.87–3.38)**	**2.51 (1.86–3.39)**
Midwife-led hospital birth NL	751	4.1 (3.6–4.6)	1.00	1.00
Alongside midwifery unit England	466	5.9 (4.7–7.0)	**1.48 (1.16–1.88)**	**1.47 (1.15–1.87)**
Obstetric unit England	1,412	16.6 (14.1–19.2)	**4.70 (3.76–5.87)**	**4.68 (3.75–5.83)**
*Transfer of care during labour or directly postpartum*				
Home NL	3,541	15.2 (14.4–16.1)	1.00	1.00
Home England	1,434	11.6 (10.4–12.7)	**0.73 (0.64–0.83)**	**0.73 (0.64–0.83)**
Freestanding midwifery unit England	562	9.2 (7.7–10.6)	**0.56 (0.47–0.68)**	**0.55 (0.45–0.67)**
Midwife-led hospital birth NL	5,426	29.4 (28.0–30.9)	1.00	1.00
Alongside midwifery unit England	1,024	12.9 (11.0–14.8)	**0.35 (0.30–0.43)**	**0.35 (0.29–0.42)**

^Weighted to reflect each unit’s separate duration of participation and probability of being sampled; confidence intervals take account of the clustered nature of the data.

* Adjusted for maternal age, gestational age, socioeconomic position and ethnic background

Rates of epidural or spinal anaesthesia varied from 16.5% to 41.9% among nulliparous and from 1.4% to 16.6% among multiparous women. For multiparous women, rates were higher among all English compared to Dutch groups. For nulliparous women rates were higher in those planning birth at home or in obstetric units compared to Dutch groups.

### Third or fourth degree perineal trauma, episiotomy and oxytocin during the third stage of labour

Rates of third or fourth degree perineal trauma varied from 3.9% to 4.9% among nulliparous and from 0.9% to 1.8% among multiparous women (Table 7). Nulliparous women who planned birth in alongside units in England had higher rates compared to Dutch women who planned midwife-led hospital births. There were no differences between other groups.

**Table 7:** Third of fourth degree perineal trauma, episiotomy and oxytocin during the third stage of labour.

Planned place of birth	No of events	Incidence/ 100 (99% CI)^	Odds ratio (99% CI)	
			Unadjusted	Adjusted*
**Nulliparous women**				
*3*^*rd*^ *or 4*^*th*^ *degree perineal trauma*				
Home NL	690	4.0 (3.6–4.4)	1.00	1.00
Home England	186	4.3 (3.4–5.2)	1.06 (0.83–1.36)	1.06 (0.82–1.36)
Freestanding midwifery unit England	202	4.0 (3.0–5.0)	1.00 (0.75–1.31)	1.07 (0.81–1.42)
Midwife-led hospital birth NL	747	3.9 (3.5–4.3)	1.00	1.00
Alongside midwifery unit England	395	4.9 (3.9–5.8)	1.26 (1.00–1.60)	**1.34 (1.05–1.69)**
Obstetric unit England	459	4.4 (3.6–5.2)	1.14 (0.92–1.40)	1.20 (0.97–1.49)
*Episiotomy*				
Home NL	6,988	40.8 (39.9–43.4)	1.00	1.00
Home England	725	15.9 (14.3–17.5)	**0.27 (0.24–0.31)**	**0.26 (0.22–0.29)**
Freestanding midwifery unit England	844	16.0 (13.1–18.9)	**0.28 (0.22–0.34)**	**0.28 (0.22–0.34)**
Midwife-led hospital birth NL	7,997	41.7 (39.9–43.4)	1.00	1.00
Alongside midwifery unit England	1,758	21.7 (18.7–24.7)	**0.39 (0.32–0.47)**	**0.39 (0.32–0.48)**
Obstetric unit England	2,978	29.2 (26.5–31.9)	**0.58 (0.50–0.67)**	**0.58 (0.49–0.68)**
*Oxytocin during the third stage of labour*				
Home NL	13,084	77.4 (75.5–79.4)	1.00	1.00
Home England	3,061	70.9 (66.8–75.0)	**0.71 (0.56–0.89)**	**0.70 (0.55–0.88)**
Freestanding midwifery unit England	4,069	79.6 (73.1–86.2)	1.14 (0.75–1.73)	1.12 (0.74–1.70)
Midwife-led hospital birth NL	15,888	83.7 (82.3–85.1)	1.00	1.00
Alongside midwifery unit England	7,058	87.1 (82.9–91.3)	1.32 (0.89–1.95)	1.29 (0.87–1.90)
Obstetric unit England	9,677	94.1 (92.5–95.8)	**3.13 (2.28–4.29)**	**3.03 (2.20–4.17)**
**Multiparous women**				
*3*^*rd*^ *or 4*^*th*^ *degree perineal trauma*				
Home NL	281	1.2 (1.0–1.4)	1.00	1.00
Home England	121	1.0 (0.7–1.3)	0.81 (0.59–1.12)	0.83 (0.59–1.16)
Freestanding midwifery unit England	50	0.9 (0.5–1.3)	0.74 (0.47–1.16)	0.75 (0.48–1.18)
Midwife-led hospital birth NL	320	1.8 (1.5–2.0)	1.00	1.00
Alongside midwifery unit England	126	1.6 (1.1–2.0)	0.88 (0.63–1.25)	0.90 (0.64–1.26)
Obstetric unit England	140	1.6 (1.2–2.1)	0.92 (0.67–1.28)	0.96 (0.71–1.30’)
*Episiotomy*				
Home NL	1,802	7.8 (7.0–8.5)	1.00	1.00
Home England	167	1.4 (1.1–1.7)	0.17 (0.13–0.22)	**0.17 (0.13–0.22)**
Freestanding midwifery unit England	135	2.3 (1.7–2.9)	0.28 (0.21–0.37)	**0.28 (0.21–0.37)**
Midwife-led hospital birth NL	2,068	11.4 (10.5–12.4)	1.00	1.00
Alongside midwifery unit England	282	3.7 (2.9–4.5)	**0.30 (0.24–0.38)**	**0.30 (0.23–0.38)**
Obstetric unit England	664	7.5 (6.2–8.7)	**0.63 (0.51–0.77)**	**0.62 (0.50–0.75)**
*Oxytocin during the third stage of labour*				
Home NL	12,859	55.8 (53.3–58.3)	1.00	1.00
Home England	8,034	68.0 (64.0–72.0)	**1.68 (1.37–2.07)**	**1.67 (1.35–2.06)**
Freestanding midwifery unit England	4,512	76.3 (68.4–84.2)	**2.55 (1.63–3.98)**	**2.49 (1.59–3.89)**
Midwife-led hospital birth NL	12,920	73.0 (71.0–75.1)	1.00	1.00
Alongside midwifery unit England	6,822	84.7 (79.8–89.5)	**2.04 (1.38–3.01)**	**1.97 (1.34–2.90)**
Obstetric unit England	8,252	93.6 (91.6–95.5)	**5.37 (3.84–7.49)**	**5.20 (3.73–7.25)**

^Weighted to reflect each unit’s separate duration of participation and probability of being sampled; confidence intervals take account of the clustered nature of the data.

* Adjusted for maternal age, gestational age, socioeconomic position and ethnic background.

Episiotomy rates varied from 15.9% to 41.7% among nulliparous and from 1.4% to 11.4% among multiparous women. Rates were lower among all groups in England compared to Dutch groups.

Rates of oxytocin during the third stage of labour varied from 70.9% to 94.1% among nulliparous and from 55.8% to 93.6% among multiparous women. Nulliparous English women were less likely to receive oxytocin if they had planned home births and more likely if they planned birth in obstetric units compared to Dutch women planning home and midwife-led hospital birth respectively. Multiparous English women were more likely to receive oxytocin in all groups compared to Dutch women.

### Additional analyses

After removing records with discrepancies in information on start of labour in midwife-led or obstetrician-led care and, secondly, after removing 4,916 Dutch women who planned midwife-led hospital birth but who gave birth at home most results were similar to the main findings (S1–S5 Tables).

From the Deliver study, 3674 women were included starting labour in midwife-led care; 2152 planned home birth and 1522 planned midwife-led hospital birth. Comparison of BPS and Deliver study data showed that English women were more likely to have higher BMIs (data not reported). When CS rates were compared between English women in the BPS and Dutch women in the Deliver study, and BMI was included as confounder, results were similar to the main findings (S6 Table).

## Discussion

Rates of CS and instrumental vaginal birth were consistently higher among English women planning obstetric unit birth compared with Dutch women planning midwife-led hospital births. When comparing midwife-led settings, rates of CS among planned births in alongside units in England were lower compared with planned midwife-led hospital births in the Netherlands for multiparous women and for nulliparous women this difference was statistically significant after excluding unplanned home births in the Netherlands. Among nulliparous women, rates of instrumental vaginal birth and operative birth were lower in English compared to Dutch midwife-led settings.

Multiparous women in both countries had similarly lower rates of CS among planned home births compared to planned midwife-led hospital births (the Netherlands) or births planned in alongside units (England). For nulliparous women planned home births were associated with lower CS rates compared to planned hospital births only in the Netherlands.

Transfer, augmentation and episiotomy rates were much lower in England compared to the Netherlands for almost all groups. In most comparisons, epidural rates were higher for the English groups. Oxytocin for the third stage was given more often in many comparison groups in England but less often among planned home births among nulliparous women.

### Strengths and limitations

The main strength of this study is that we combined the two largest studies on planned home and midwife-led hospital birth in high income countries into one dataset. This enabled us to gain insight into differences in care processes and outcomes between comparable groups.

This study has some limitations. Although we compared similar planned places of birth, some characteristics will be different. For example, obstetricians in the Netherlands have no role in uncomplicated planned midwife-led hospital births in the Netherlands whereas in England obstetricians are responsible for low risk births planned in obstetric units even if midwives provide most of the care.

Although criteria for assessing suitability of planned out of hospital birth in England and the Netherlands are rather similar, some differences exist.[31,32] Women in both countries planning birth in hospital may have had higher rates of unknown risk factors due to self-selection by women themselves or midwives.[28] The English NICE guideline lists risk factors that require individual assessment with regard to planned place of birth, such as BMI 30–34 kg/m^2^.[31] In addition, women planning birth in obstetric units in England more often had complicating conditions at the start of care in labour compared to other BPS subgroups.[6] Therefore, differences found between groups should be interpreted with caution.

In the Netherlands information on BMI is not recorded in the perinatal database. Nevertheless, sensitivity analysis using data from the Deliver study to be able to control for BMI showed similar results as the main analysis. Dutch data came from routine perinatal registrations and information for some women was missing, for example on planned place of birth, and some data would have been misclassified, for example on whether labour started in primary or secondary care.[28] Finally, no distinction could be made in the Birthplace study data between epidural analgesia for pain relief and spinal analgesia for CS and therefore we combined these two in the Netherlands as well. Some differences found in the rate of regional anaesthesia and episiotomy rates will be due to differences in CS, in particular when births planned in obstetric units in England were compared with Dutch planned hospital births. However, differences in rates of these outcomes were so large that this cannot be explained by differences in CS rate alone.

### Interpretation

Lower rates of CS among planned home births compared to planned midwife-led hospital birth for multiparous women in both countries and for nulliparous women in the Netherlands confirm findings in other studies.[22,26,38,39] If home birth is uncommon, women planning birth at home may be a selective group and be very motivated to avoid medical interventions.[22] In that light it is surprising that lower CS rates were found among planned home births compared with planned midwife-led hospital births for nulliparous women in the Netherlands but not in England. The relatively low number of home births in England may have limited power to find significant differences. Another explanation may be that transfer rates for nulliparous women were lower among planned home births compared to planned hospital births in the Netherlands but not among planned births at home versus in alongside units in England. Another Dutch study showed that rising transfer rates over a period of nine years were associated with increased CS rates among nulliparous women.[40]

Transfer rates during and immediately after labour were much lower for all comparison groups in England compared to the Netherlands. This is consistent with a systematic review in which the Netherlands had the highest transfer rates during labour of 14 countries.[41] Request for pain relief is an important reason for transfer of care. Rates of regional analgesia were much higher in England, and therefore higher transfer rates might have been expected. However, other types of pain medication, such as inhaled analgesia or opioids, can be administered in midwifery units and at home in England but not in the Netherlands. Request for pain medication was the main reason for transfer before birth in a Dutch study (30.5%)[42] whereas this was the primary reason for only 13.0% of transferred women in the BPS.[43] Only recently, inhaled analgesia has been re-introduced in a few newly set up midwifery units in the Netherlands, but this was not available in 2009. Changes are being planned in the maternity care system that will enable primary care midwives to continue looking after women in midwifery units not currently eligible for midwife-led care, such as those in need of pain medication, by expanding their scope of practice.[42] It is likely that this will lead to lower transfer rates. Another reason for the higher transfer rates may be the high case load of Dutch midwives, which is currently 105 care units a year; one care unit equals the amount paid for complete midwifery care during pregnancy, birth and the postpartum period for one woman. [44] This makes it difficult for midwives to provide continuous support throughout the active stage of labour. Further research is needed to examine ways to reduce avoidable transfers of care in the Netherlands.

Rates of labour augmentation and instrumental vaginal births might have been expected to be higher in England in view of high regional anaesthesia rates[45] but they were actually much lower in most English subgroups. Augmentation of labour rates were lower in all groups, although not significantly so in women with birth planned in obstetric units. This raises questions about possible differences in management of progress of labour when women have epidural analgesia in both countries. Lower rates of instrumental vaginal births among nulliparous English women with birth planned in midwife-led care were offset by higher rates among all women with planned obstetric unit birth and may be associated with lower transfer rates in England.

Episiotomy rates were much lower in all English groups compared to those in the Netherlands and for most women this was not balanced by a higher risk of third or fourth degree tears. A Dutch study in primary care showed that prolonged second stage of labour was an important reason for performing episiotomy but low rates of prolonged second stage in that study suggest that this indication may be used too often.[46] More research is needed into strategies to reduce episiotomy rates in the Netherlands.

Rates of most obstetric interventions were highest among English women who planned birth in obstetric units. Some of this may be explained by selection bias; the fact that intervention rates in obstetric units are higher in areas with more provision of midwife-led care may indicate that women in obstetric units in these areas have a different risk profile compared to women in midwife-led units.[35] Nevertheless, even if possible selection bias is taken into account, it is unlikely that unmeasured risk differences would completely account for this. Although midwives provide most of the care in uncomplicated births in obstetric units, women appear to be more likely to receive obstetric care than those planning birth in midwife-led settings. Other studies have shown higher rates of obstetric interventions in shared care or obstetrician led models of care compared to midwife-led continuity of care.[21] Although midwifery units in England may not provide continuity of care if complications arise, they do provide midwife-led care. Our findings support recommendations from the English guideline on intrapartum care, i.e. that giving birth at home or in a midwifery unit is particularly suitable for multiparous women and giving birth in a midwifery unit for nulliparous women.[47] In addition, it is important to develop strategies to reduce rates of medical interventions among low risk births in obstetric units and to increase the proportion of women in England planning birth in midwife-led settings.

## Conclusion

When considering maternal outcomes, our findings confirm benefits of planning birth in midwife-led settings for low risk women. Further research is needed into strategies to decrease rates of medical interventions in obstetric units in England. More evidence is required on ways to reduce avoidable rates of transfer, augmentation of labour after transfer and episiotomy in births planned in midwife-led settings in the Netherlands among low risk women.

## Supporting information

S1 Table Planned place of birth and rate of caesarean section after exclusion of Dutch women with conflicting information on start labour in midwife-led or obstetrician-led care at the onset of labour. (DOCX)

S2 Table Planned place of birth and rate of instrumental vaginal birth (ventouse or forceps) after exclusion of Dutch women with conflicting information on start labour in midwife-led or obstetrician-led care at the onset of labour. (DOCX)

S3 Table Planned place of birth and rate of operative births (caesarean section or instrumental vaginal birth) after exclusion of Dutch women with conflicting information on start labour in midwife-led or obstetrician-led care at the onset of labour. (DOCX)

S4 Table Comparison of difference in CS rate between planned home birth and planned hospital birth in the Netherlands with the difference in CS rate between planned home and planned birth in an alongside midwifery unit in England, after exclusion of Dutch women with conflicting information on start labour in midwife-led or obstetrician-led care at the onset of labour. (DOCX)

S5 Table Planned place of birth and rate of caesarean section after exclusion of women with unplanned homebirths in the Netherlands. (DOCX)

S6 Table Planned place of birth and rate of caesarean section reporting data of the DELIVER and BPS studies including adjustment for BMI. (DOCX)
