# Supplementary material for: Mode of birth and medical interventions among women at low risk of complications: A cross-national comparison of birth settings in England and the Netherlands
Source: PLoS One. 2017 Jul 27;12(7):e0180846. doi: 10.1371/journal.pone.0180846 (PMC5531544; doi:10.1371/journal.pone.0180846)
Supplement: S1 Table — (DOCX) [file pone.0180846.s001.docx]

**Table S1: Planned place of birth and rate of caesarean section after exclusion of Dutch women with conflicting information on start labour in midwife-led or obstetrician-led care at the onset of labour**

| **Planned place of birth** | **No of events/ birth** | **Incidence of caesarean section**  **/ 100^ (95% CI)** | **Odds ratio (95% CI)** | |
| --- | --- | --- | --- | --- |
|  |  |  | **Unadjusted** | **Adjusted*** |
| **Nulliparous women** |  |  |  |  |
| Home NL | 916 | 6.2 (5.7- 6.6) | 1.00 | 1.00 |
| Home England | 355 | 8.4 (7.1-9.7) | **1.39 (1.16-1.67)** | **1.28 (1.06-1.54)** |
| Freestanding midwifery unit England | 345 | 6.5 (5.5-7.5) | 1.06 (0.89-1.26) | 1.13 (0.95-1.34) |
|  |  |  |  |  |
| Midwife-led hospital birth NL | 1,253 | 7.9 (7.2-8.4) | 1.00 | 1.00 |
| Alongside midwifery unit England | 619 | 7.6 (6.5-8.8) | 0.97 (0.81-1.16) | 1.01 (0.83-1.24) |
| Obstetric unit England | 1,575 | 15.5 (13.9-17.1) | **2.15 (1.86-2.49)** | **2.24 (1.93-2.60)** |
| **Multiparous women** |  |  |  |  |
| Home NL | 145 | 0.7 (0.5-0.8) | 1.00 | 1.00 |
| Home England | 80 | 0.6 (.5-0.8) | 0.95 (0.70-1.30) | 0.93 (0.68-1.28) |
| Freestanding midwifery unit England | 44 | 0.7 (0.5- 0.9) | 1.10 (0.76- 1.59) | 1.12 (0.77-1.63) |
|  |  |  |  |  |
| Midwife-led hospital birth NL | 186 | 1.1 (0.9-1.3) | 1.00 | 1.00 |
| Alongside midwifery unit England | 87 | 1.0 (0.7-1.3) | 0.93 (0.65-1.32) | 0.92 (0.63-1.34) |
| Obstetric unit England | 446 | 5.1 (4.1-6.1) | **4.79 (3.69-6.23)** | **4.84 (3.76-6.21)** |

^Weighted to reflect each unit’s separate duration of participation and probability of being sampled; confidence intervals take account of the clustered nature of the data.

* Adjusted for maternal age, gestational age, socioeconomic position and ethnic background.
