# Supplementary material for: Mode of birth and medical interventions among women at low risk of complications: A cross-national comparison of birth settings in England and the Netherlands
Source: PLoS One. 2017 Jul 27;12(7):e0180846. doi: 10.1371/journal.pone.0180846 (PMC5531544; doi:10.1371/journal.pone.0180846)
Supplement: S2 Table — (DOCX) [file pone.0180846.s002.docx]

**Table S2: Planned place of birth and rate of instrumental vaginal birth (ventouse or forceps) after exclusion of Dutch women with conflicting information on start labour in midwife-led or obstetrician-led care at the onset of labour**

| **Planned place of birth** | **No of events/ birth** | **Incidence of instrumental vaginal birth/**  **100 (95% CI)^** | **Odds ratio (95% CI)** | |
| --- | --- | --- | --- | --- |
|  |  |  | **Unadjusted** | **Adjusted*** |
| **Nulliparous women** |  |  |  |  |
| Home NL | 2,254 | 15.2 (14.4- 15.9) | 1.00 | 1.00 |
| Home England | 571 | 12.3 (11.3-13.5) | **0.79 (0.70- 0.89)** | **0.72 (0.64-0.80)** |
| Freestanding midwifery unit England | 602 | 10.7 (9.0-12.5) | **0.67 (0.56- 0.81)** | **0.68 (0.57-0.82)** |
|  |  |  |  |  |
| Midwife-led hospital birth NL | 2,639 | 16.6 (15.8- 17.4) | 1.00 | 1.00 |
| Alongside midwifery unit England | 1,289 | 16.0 (14.0- 17.9) | 0.96 (0.82- 1.12) | 0.96 (0.81-1.13) |
| Obstetric unit England | 2,251 | 22.5 (19.9- 25.1) | **1.46 (1.25- 1.72)** | **1.47 (1.24-1.74)** |
| **Multiparous women** |  |  |  |  |
| Home NL | 182 | 0.8 (0.7-0.9) | 1.00 | 1.00 |
| Home England | 109 | 0.9 (0.7-1.1) | 1.09 (0.85- 1.41) | 1.10 (0.85-1.42) |
| Freestanding midwifery unit England | 69 | 1.0 (0.7-1.3) | 1.24 (0.87- 1.77) | 1.22 (0.85-1.37) |
|  |  |  |  |  |
| Midwife-led hospital birth NL | 281 | 1.7 (1.5- 1.9) | 1.00 | 1.00 |
| Alongside midwifery unit England | 188 | 2.4 (1.9- 3.0) | **1.46 (1.14- 1.89)** | **1.45 (1.12-1.88)** |
| Obstetric unit England | 491 | 5.7 (4.8- 6.6) | **3.53 (2.86- 4.35)** | **3.49 (2.82-4.32)** |

^Weighted to reflect each unit’s separate duration of participation and probability of being sampled; confidence intervals take account of the clustered nature of the data.

* Adjusted for maternal age, gestational age, socioeconomic position and ethnic background.
