# Supplementary material for: Mode of birth and medical interventions among women at low risk of complications: A cross-national comparison of birth settings in England and the Netherlands
Source: PLoS One. 2017 Jul 27;12(7):e0180846. doi: 10.1371/journal.pone.0180846 (PMC5531544; doi:10.1371/journal.pone.0180846)
Supplement: S3 Table — (DOCX) [file pone.0180846.s003.docx]

**Table S3: Planned place of birth and rate of operative births (caesarean section or instrumental vaginal birth) after exclusion of Dutch women with conflicting information on start labour in midwife-led or obstetrician-led care at the onset of labour**

| **Planned place of birth** | **No of events/ birth** | **Incidence of operative births/**  **100^ (95% CI)** | **Odds ratio (95% CI)** | |
| --- | --- | --- | --- | --- |
|  |  |  | **Unadjusted** | **Adjusted*** |
| **Nulliparous women** |  |  |  |  |
| Home NL | 3,170 | 21.4 (20.5- 22.2) | 1.00 | 1.00 |
| Home England | 926 | 20.8 (19.1- 22.5) | 0.97 (0.86-1.08) | **0.87 (0.78-0.97)** |
| Freestanding midwifery unit England | 947 | 17.2 (15.0- 19.5) | **0.77 (0.65-0.90)** | **0.79 (0.68-0.93)** |
|  |  |  |  |  |
| Midwife-led hospital birth NL | 3,892 | 24.4 (23.4- 25.5) | 1.00 | 1.00 |
| Alongside midwifery unit England | 1,908 | 23.6 (21.2- 26.0) | 0.95 (0.83-1.10) | 0.98 (0.83-1.15) |
| Obstetric unit England | 3,826 | 38.3 (35.4-40.7) | **1.90 (1.67-2.15)** | **1.97 (1.74-2.23)** |
| **Multiparous women** |  |  |  |  |
| Home NL | 327 | 1.5 (1.3- 1.6) | 1.00 | 1.00 |
| Home England | 189 | 1.5 (1.3- 1.8) | 1.03 (0.84-1.26) | 1.03 (0.83-1.26) |
| Freestanding midwifery unit England | 113 | 1.7 (1.3- 2.2) | 1.18 (0.88-1.59) | 1.18 (0.87-1.61) |
|  |  |  |  |  |
| Midwife-led hospital birth NL | 467 | 2.8 (2.5- 3.1) | 1.00 | 1.00 |
| Alongside midwifery unit England | 275 | 3.5 (2.7- 4.2) | 1.25 (0.99-1.59) | 1.24 (0.97-1.60) |
| Obstetric unit England | 937 | 10.8 (9.4- 12.2) | **4.21 (3.52-5.05)** | **4.22 (3.55-5.03)** |

^Weighted to reflect each unit’s separate duration of participation and probability of being sampled; confidence intervals take account of the clustered nature of the data.

* Adjusted for maternal age, gestational age, socioeconomic position and ethnic background.
