# Supplementary material for: Mode of birth and medical interventions among women at low risk of complications: A cross-national comparison of birth settings in England and the Netherlands
Source: PLoS One. 2017 Jul 27;12(7):e0180846. doi: 10.1371/journal.pone.0180846 (PMC5531544; doi:10.1371/journal.pone.0180846)
Supplement: S4 Table — (DOCX) [file pone.0180846.s004.docx]

**Table S4. Comparison of difference in CS rate between planned home birth and planned hospital birth in the Netherlands with the difference in CS rate between planned home and planned birth in an alongside midwifery unit in England**, **after exclusion of Dutch women with conflicting information on start labour in midwife-led or obstetrician-led care at the onset of labour**

| **Planned place of birth** | **No of events/ birth** | **Incidence of caesarean section**  **/ 100^ (95% CI)** | **Odds ratio (95% CI)** | |
| --- | --- | --- | --- | --- |
|  |  |  | **Unadjusted** | **Adjusted*** |
| **Nulliparous women** |  |  |  |  |
| Midwife-led hospital birth NL | 1,253 | 7.9 (7.3- 8.4) | 1.00 | 1.00 |
| Home NL | 916 | 6.2 (5.7- 6.6) | **0.77 (0.70- 0.85)** | **0.83 (0.75-0.92)** |
| **Multiparous women** |  |  |  |  |
| Midwife-led hospital birth NL | 186 | 1.1 (0.9- 1.3) | 1.00 | 1.00 |
| Home NL | 145 | 0.6 (0.5- 0.8) | **0.59 (0.4- 0.74)** | **0.66 (0.52-0.84)** |

^Weighted to reflect each unit’s separate duration of participation and probability of being sampled; confidence intervals take account of the clustered nature of the data.

* Adjusted for maternal age, gestational age, socioeconomic position and ethnic background.
