# Supplementary material for: Mode of birth and medical interventions among women at low risk of complications: A cross-national comparison of birth settings in England and the Netherlands
Source: PLoS One. 2017 Jul 27;12(7):e0180846. doi: 10.1371/journal.pone.0180846 (PMC5531544; doi:10.1371/journal.pone.0180846)
Supplement: S5 Table — (DOCX) [file pone.0180846.s005.docx]

**Table S5: Planned place of birth and rate of caesarean section after exclusion of women with unplanned homebirths in the Netherlands**

| **Planned place of birth** | **No of events** | **Incidence of caesarean section**  **/ 100^ (95% CI)** | **Odd ratio (95% CI)** | |
| --- | --- | --- | --- | --- |
|  |  |  | **Unadjusted** | **Adjusted*** |
| **Nulliparous women** |  |  |  |  |
| Midwife-led hospital birth NL | 1,806 | 10.2 (9.6-10.8) | 1.00 | 1.00 |
| Alongside midwifery unit England | 619 | 7.6 (6.5-8.8) | **0.73 (0.61-0.86)** | **0.77 (0.64-0.94)** |
| Obstetric unit England | 1,575 | 15.5 (13.9-17.1) | **1.61 (1.40-1.85)** | **1.71 (1.48-1.97)** |
| **Multiparous women** |  |  |  |  |
| Midwife-led hospital birth NL | 271 | 1.8 (1.5-2.0) | 1.00 | 1.00 |
| Alongside midwifery unit England | 87 | 1.0 (0.7-1.3) | **0.58 (0.41-0.81)** | **0.58 (0.40-0.84)** |
| Obstetric unit England | 446 | 5.1 (4.1-6.1) | **3.00 (2.35-3.83)** | **3.06 (2.42-3.87)** |

^Weighted to reflect each unit’s separate duration of participation and probability of being sampled; confidence intervals take account of the clustered nature of the data.

* Adjusted for maternal age, gestational age, socioeconomic position and ethnic background.
