# Supplementary material for: Mode of birth and medical interventions among women at low risk of complications: A cross-national comparison of birth settings in England and the Netherlands
Source: PLoS One. 2017 Jul 27;12(7):e0180846. doi: 10.1371/journal.pone.0180846 (PMC5531544; doi:10.1371/journal.pone.0180846)
Supplement: S6 Table — (DOCX) [file pone.0180846.s006.docx]

**Table S6. Planned place of birth and rate of caesarean section reporting data of the DELIVER and BPS studies including adjustment for BMI**

| **Planned place of birth** | **Total number** | **No of events** | **Incidence of caesarean section**  **/ 100** ^ **(95% CI)** | **Odds ratio (95% CI)** | |
| --- | --- | --- | --- | --- | --- |
|  |  |  |  | **Unadjusted** | **Adjusted*** |
| **Nulliparous women** |  |  |  |  |  |
| Home NL | 940 | 69 | 7.4 (5.7-9.0) | 1.00 | 1.00 |
| Home England | 4,400 | 351 | 8.0 (7.2-8.8) | 1.14 (0.84-1.54) | 1.04 (0.77-1.41) |
| Freestanding midwifery unit England | 5,115 | 345 | 6.7 (6.1-7.4) | 0.93 (0.69-1.26) | 0.99 (0.73-1.35) |
|  |  |  |  |  |  |
| Midwife-led hospital birth NL | 766 | 73 | 9.5 (7.4-11.6) | 1.00 | 1.00 |
| Alongside midwifery unit England | 8,174 | 618 | 7.6 (7.0-8.1) | 0.78 (0.58-1.07) | 0.84 (0.62-1.16) |
| Obstetric unit England | 10,254 | 1,571 | 15.3 (14.6-16.0) | **1.80 (1.33-2.44)** | **1.94 (1.42-2.64)** |
| **Multiparous women** |  |  |  |  |  |
| Home NL | 1,212 | 10 | 0.8 (0.3-1.3) | 1.00 | 1.00 |
| Home England | 11,988 | 80 | 0.7 (0.5-0.8) | 0.81 (0.41-1.57) | 0.79 (0.40-1.56) |
| Freestanding midwifery unit England | 6,003 | 44 | 0.7 (0.5-0.9) | 0.89 (0.44-1.77) | 0.90 (0.44-1.81) |
|  |  |  |  |  |  |
| Midwife-led hospital birth NL | 756 | 15 | 2.0 (1.0-3.0) | 1.00 | 1.00 |
| Alongside midwifery unit England | 8,179 | 85 | 1.0 (0.8-1.3) | **0.48 (0.26-0.89)** | **0.49 (0.27-0.91)** |
| Obstetric unit England | 8,792 | 444 | 5.1 (4.6-5.5) | **2.65 (1.49-4.70)** | **2.69 (1.51-4.78)** |

^ All reported confidence intervals take account of the clustered nature of the data.

** Adjusted for BMI maternal age, gestational age, socioeconomic position and ethnic background. Missing data for BMI were not included in total number; in the Netherlands n=70, in England n=212. Women for whom BMI was not recorded were classified as a separate group; in the Netherlands n=85, in England n=11,325.
